# Combination of Porous Silk Fibroin Substrate and Gold Nanocracks as a Novel SERS Platform for a High-Sensitivity Biosensor

**DOI:** 10.3390/bios11110441

**Published:** 2021-11-06

**Authors:** Ji Hyeon Choi, Munsik Choi, Taeyoung Kang, Tien Son Ho, Seung Ho Choi, Kyung Min Byun

**Affiliations:** 1 Department of Biomedical Engineering, Kyung Hee University, Yongin 17104, Korea; jihyeon_n1@naver.com; 2 Department of Electronics and Information Convergence Engineering, Kyung Hee University, Yongin 17104, Korea; terrykang95@khu.ac.kr; 3 Medical Device R&D Center, Seoul National University Bundang Hospital, Seongnam 13695, Korea; 99529@snubh.org; 4 Department of Biomedical Engineering, Yonsei University, 1 Yonseidae-gil, Wonju 26493, Korea; hotienson@yonsei.ac.kr

**Keywords:** silk fibroin, porous, gold nanocracks, surface-enhanced Raman scattering, sensitive, overlap integral

## Abstract

Novel concepts for developing a surface-enhanced Raman scattering (SERS) sensor based on biocompatible materials offer great potential in versatile applications, including wearable and in vivo monitoring of target analytes. Here, we report a highly sensitive SERS sensor consisting of a biocompatible silk fibroin substrate with a high porosity and gold nanocracks. Our silk-based SERS detection takes advantage of strong local field enhancement in the nanoscale crack regions induced by gold nanostructures evaporated on a porous silk substrate. The SERS performance of the proposed sensor is evaluated in terms of detection limit, sensitivity, and linearity. Compared to the performance of a counterpart SERS sensor with a thin gold film, SERS results using 4-ABT analytes present that a significant improvement in the detection limit and sensitivity by more than 4 times, and a good linearity and a wide dynamic range is achieved. More interestingly, overlap is integral, and a quantitative measure of the local field enhancement is highly consistent with the experimental SERS enhancement.

## 1. Introduction

Surface-enhanced Raman scattering (SERS) has been widely employed as a spectroscopic technique for the identification of chemical and biological species due to its advantages of high sensitivity, unique spectroscopic fingerprints, and non-destructive detection [1,2,3]. SERS can greatly enhance weak Raman signals that are often overwhelmed by Rayleigh scattering and fluorescence background noise. The principal amplification of SERS arises from an enhancement of local electromagnetic fields near or at a nanostructured metallic substrate, which is associated with the resonant excitation of localized surface plasmons [4,5]. It is now undoubtedly acknowledged that SERS signals are amplified by the molecules located in nanogaps where electromagnetic fields are largely confined, which are called hot spots [6,7]. Those plasmonic localization modes are extremely sensitive to subtle nanoscale perturbations in the surrounded medium [8].

Together with noble metals, in-depth studies on the surface enhancement of organic and inorganic materials have been conducted to produce a strong SERS effect. For example, conjugated polymers (CPs) as organic macromolecules can combine the high conductivity of metals with the chemical tunability of polymers. Such CP-based detection systems are very sensitive to minor perturbations and allow a dramatic improvement of the detection limit compared to a biosensing scheme based on small molecule sensor elements [9]. A single analyte interacts with multiple monomers of the CP through the conjugated π-electron system, leading to a great signal amplification effect in CP-based biosensors using an optical transducer [10]. Additionally, inorganic semiconductor materials, such as metal oxides, achieved an enhanced Raman signals via a doped semiconductor by changing the doping species and ion content [11]. Their SERS effect is mainly associated with a chemical enhancement, and thus, the enhancement factor is relatively low due to a lack of plasmon resonances in the conduction bands [12].

Recently, biological and biocompatible materials have attracted considerable attention towards diagnostic devices in various fields, ranging from medical and physiological monitoring to defense applications [13,14,15]. Although those advances are promising, they typically require complex nanofabrication, delicate assembly, and sophisticated detection [16,17]. To our knowledge, no one has yet demonstrated that naturally occurring biomaterials can be used as a versatile nanomaterial platform possessing highly sensitive, cost-effective, and easily customized detection abilities. Specifically, nanostructures or nanomaterials in nature allow for intense light–matter interactions, which is useful for realizing a highly sensitive detection system.

Silk fibroin material has gained much interest due to its advantageous characteristics, such as biocompatibility, biodegradability, and no inflammatory response in vivo [18,19,20]. A single silk filament with a diameter of ~20 µm contains 3000~4000 individual nanofibrils whose size distribution ranges from 30 to 200 nm. Nearly parallel nanofibrils run along the longitudinal axis of the filament. Unique hierarchical silk nanostructures of a large surface-to-volume ratio can enhance the scattering power [21].

However, conventional approaches to obtain silk-based plasmonic devices often suffer from expensive and harsh fabrication processes [22,23]. Since free-standing biological films tend to be mechanically weak and thermally unstable, the use of rigid substrates to support a thin silk film and to transfer a nanoscale pattern is unavoidable. During the transfer printing process, which is the most common technique in patterning biopolymers, the exposure of silk material to high temperature, high pressure, and highly reactive chemical solutions could be harmful to preserving its biocompatibility and biofunctionality [24,25].

In this study, we developed a simple but effective fabrication process for a silk substrate and implemented an enhanced SERS sensor through a combination of a silk surface with a high porosity and gold nanostructures. We confirmed that a silk-based porous SERS substrate provided an enhanced detection limit and enhanced sensitivity as well as fairly good linearity. Without the assistance of rigid or soft substrates, a free-standing SERS sensor platform with gold nanocracks was successfully realized in a large area. To investigate the influence of the porous silk-based sensor on the quality of SERS signals, we performed spectroscopic experiments for several types of SERS platforms. A quantitative comparison of the detection performance, detection limit, sensitivity, linearity, and dynamic range for individual samples was also explored. It is expected that our silk-based SERS sensor has the potential to detect subtle nanoscale environmental changes with high sensitivity towards wearable and in vivo monitoring devices.

## 2. Materials and Methods

Distilled water (H_2_O) and ethyl alcohol (C_2_H_5_OH) were obtained from DaeJung Chemicals & Metals Co., LTD. (Siheung, Korea). Sodium carbonate (Na_2_CO_3_), sodium oleate (C_18_H_33_NaO_2_), calcium chloride (CaCl_2_), and 4-aminobenzenethiol (4-ABT, C_6_H_7_NS) were purchased from Sigma-Aldrich (St. Louis, MO, USA). *Bombyx mori* cocoons were supported by the Rural Development Administration (Wanju, Korea).

The fabrication procedure for a highly porous silk substrate to realize a SERS sensor is as follows: To remove the gum-like sericin layer from the silk fibroin, a native silk cocoon of *Bombyx mori* was boiled at 95 °C for 60 min in DI water with 2 mM sodium carbonate and 1.5 mM sodium oleate. Then, it was rinsed with DI water for 30 min. The degummed silk was dried in the oven at 600 °C overnight. Polyurethane (PU) was completely dissolved in formic acid to prepare 1 wt% of PU solution, and then 15 wt% of the dried silk fibroin was added to the solution. Then, 4 wt% of calcium chloride (CaCl_2_) was put into the blend solution of the silk and PU to dissolve the silk fibroin, and the solution was left stirring overnight at room temperature to obtain a homogenous solution. The silk solution was boiled until it turned yellow in color, and it was then filtered by a Mira cloth membrane. To completely remove the calcium chloride from the silk solution, the solution was poured into a dialyze tube and was kept for 2 days in a water bath. Centrifugation at 4000 RPM for 20 min was carried out to increase the concentration of the silk solution and to reduce the drying time. A drying plate was cleaned with ethanol and DI water to remove residual impurities. The centrifuged silk solution was then dried for 24 h at room temperature in a clean hood. Finally, a highly porous silk substrate with a size of 5.0 × 5.0 × 1.0 mm^3^ was obtained.

For the comparison study, several types of SERS sensors based on a silicon wafer, BK7 glass, and a porous silk substrate were prepared. First, when a 40 nm thick gold layer was applied on individual substrates at a deposition rate of 1.0 Å/s, a smooth and thin gold film was obtained for the silicon wafer and glass substrates, while a gold film on a silk substrate presented a corrugated surface profile with a roughness of a few tens of nanometers. Second, a 3 nm thick gold layer was deposited onto the silicon wafer and silk substrate to form a pattern of gold nanostructures. Under the process condition of an electron-beam evaporator (UEE, Ultech, Daegu, Korea) at a deposition rate of 0.2 Å/s, 3 nm thick gold deposition was chosen as the optimum due to it having the highest SERS intensity in our previous study [26].

We investigated the detection performance of the SERS sensors in terms of the detection limit, sensitivity, and linearity and compared with that of the SERS substrates on a silicon wafer. For this purpose, a 4-ABT analyte was used as a Raman probe molecule because the thiol group in 4-ABT allows a specific binding with gold. The SERS substrates were immersed for 15 min in various concentrations of 4-ABT solution. After 4-ABT immobilization, the substrates were rinsed with ethyl alcohol and deionized water for 5 min for non-immobilized 4-ABT removal. A confocal Raman microscope system consists of a microscope with a 100x objective lens (NA = 0.8, BX43, Olympus, Tokyo, Japan), a 785 nm laser source (I0785MM0350MF, Innovative Photonic Solutions, Monmouth Junction, NJ, USA), a Czerny–Turner spectrograph (SR-303i-A, Andor Technology, Belfast, UK), and a low dark current deep-depletion CCD (iVac, Andor Technology, Belfast, UK). SERS signals were measured at five different points for each sample within the range of 500 to 2000 cm^−1^, with a resolution of 1 cm^−1^ and an acquisition time of 50 s. After the SERS measurements, the instrument and CCD noise signals were simply subtracted from the raw SERS signals. Then, the Savitzky–Golay smoothing and polynomial baseline correction were performed sequentially.

## 3. Results and Discussion

**Figure 1:**
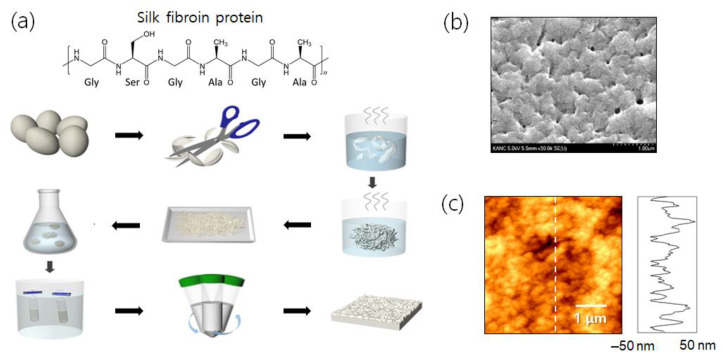
A highly porous free-standing silk substrate for SERS sensor application. (**a**) Procedures for fabricating a porous silk substrate. (**b**) FE-SEM image of the fabricated silk sample with a high porosity. (**c**) AFM image of the silk substrate and its surface profile.

In Figure 1, field emission scanning electron microscope (FE-SEM) and atomic force microscope (AFM) images were employed to investigate the surface morphological features of the fabricated porous silk substrate. After a simple yet effective chemical procedure in Figure 1a, a highly porous surface profile was successfully realized, as presented in Figure 1b. The rough and porous silk surface is clearly shown from the AFM data in Figure 1c. The silk substrate had quite rough surface features, and its average roughness was determined to be 38.55 nm. While the results are not shown, the surface roughness of the silicon wafer was as smooth as 0.15 nm. Such rough and porous silk characteristic could contribute to an excitation of localized plasmons in the presence of gold nanostructures, thereby leading to enhanced Raman scattering.

**Figure 2:**
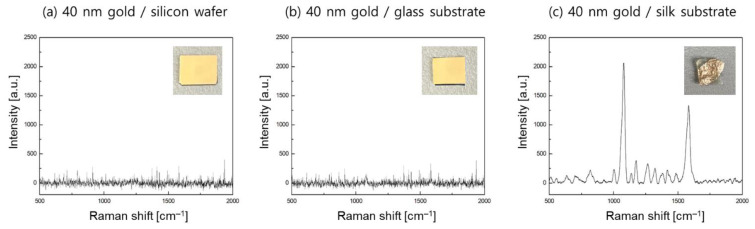
SERS experiments of 4-ABT analytes at a concentration of 10 μM for (**a**) a 40 nm thick gold film on a silicon wafer, (**b**) a 40 nm thick gold film on a BK7 glass, and (**c**) a 40 nm thick gold film on a porous silk substrate.

First, we deposited a 40 nm thick gold film onto the silk substrate to verify the effect of porous surface on the local field enhancement. For comparison study, we prepared a silicon wafer and a BK-7 glass substrate and evaporated a thin gold film at a deposition rate of 1.0 Å/s. Figure 2 showed the photo images and Raman spectra of the three samples. From the SERS experiments with a 10 μM 4-ABT Raman probe molecule, no Raman peak was found for the cases of the silicon and glass substrates because non-localized plasmon modes with a thin gold film could not contribute to an enhancement of the Raman signals to detect the 4-ABT molecules. On the other hand, a gold film on a silk substrate presented the highest peak intensity of 2042 at 1076 cm^−1^ of 4-ABT, which was assigned to the a_1_-type vibrational mode [27]. The difference in the SERS data seemed to be associated with local field excitation. From previous publications [28], when the roughness of a plasmonic substrate was larger than 5 nm, excited plasmons were strongly scattered and moved with increased disorder, which is less like a propagating wave. Specifically, for very rough surfaces, a significant change in the dispersion relation was observed because an accumulation of the electromagnetic fields resulted in highly enhanced and localized plasmons. Thus, it is expected that the rough and porous surface features of silk substrates would play a key role in acquiring enhanced Raman signals.

**Figure 3:**
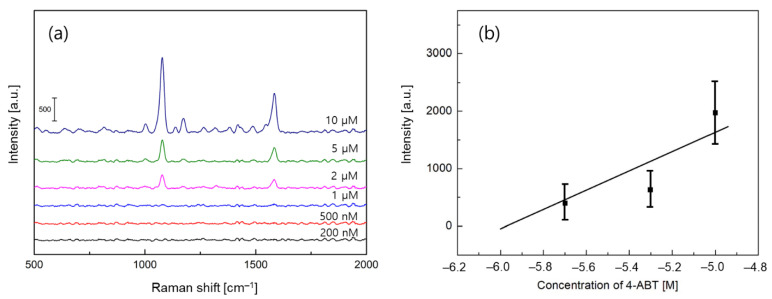
SERS sensor performance measurements. (**a**) SERS experiments of 4-ABT analytes at a varied concentration for a 40 nm thick gold film on a silk substrate. (**b**) Linear regression analysis of the Raman intensities at 1076 cm^−1^ to quantify the sensitivity and linearity.

Subsequently, we analyzed the detection limit and sensitivity performance by varying the concentration of 4-ABT in the range of 200 nM to 10 µM. For the silk sample with a thin gold film, we found that the primary SERS peak at the concentration as small as 2 µM in Figure 3a. Additionally, we calculated the slope between the intensity of the primary SERS peak and the concentration of 4-ABT using a linear regression analysis to quantify the sensitivity. In Figure 3b, the sensitivity and *R*^2^ values were determined to be 1349 and 0.71, respectively, where *R*, the coefficient of determination, was the percentage value of the dependent variable variation.

**Figure 4:**
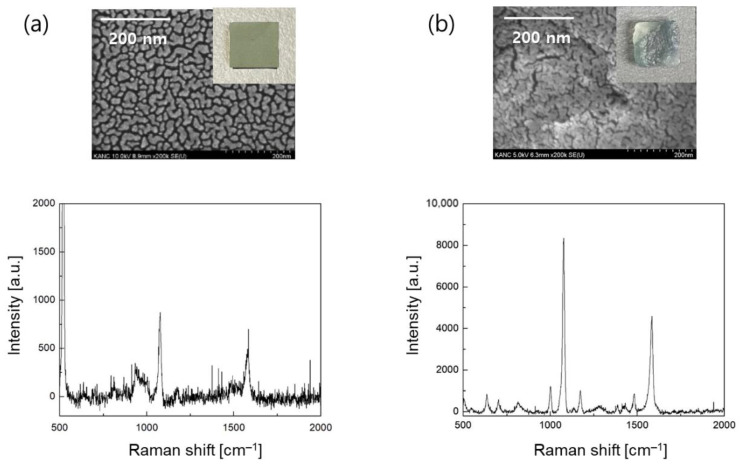
SERS sensor platform with a gold nanostructure. (**a**) FE-SEM image of a silicon wafer with gold nanostructures with a thickness of 3 nm and SERS experiments of 4-ABT analytes at a concentration of 10 μM. The sharp and intense Raman peak at 520 cm^−1^ originated from the crystalline silicon wafer. (**b**) FE-SEM image of a silk-based SERS sensor with gold nanocracks on a porous silk substrate and SERS experiments of 4-ABT analytes at a concentration of 10 μM.

Second, in order to significantly improve the SERS effect, gold nanostructures were applied onto smooth the silicon wafer and porous silk substrates. When the two substrates were loaded into the evaporation chamber, a 3 nm gold layer was deposited, and the contrast in the fabrication results was presented in Figure 4. In the case of the silicon wafer, the gold nanostructures were regularly patterned, and an average gap distance of 10.2 nm was measured between them. However, for a rough and porous silk substrate, the gold nanostructures that were obtained were fairly different from those obtained in the case of the silicon substrate. From the SEM images, we were able to find sharp cracks and fractures on the surface, and such patterns seemed to be more appropriate for the SERS effect according to the concept of hot spots, which occur at nanogaps or nanocracks where the electromagnetic fields are largely confined. For several silk samples, the gap distance of gold nanocracks was averaged to be 3.1 nm.

SERS experiments with 10 μM 4-ABT analytes demonstrated that gold nanostructures on a silicon wafer presented the highest peak value of 878 at 1076 cm^−1^, whereas gold nanocracks on a porous silk substrate showed a highly enhanced Raman signal of 8350. Note that in in Figure 4a, the sharp and intense Raman peak at 520 cm^−1^ was obtained from crystalline silicon, which was not found in Figure 2a, as the entire silicon surface was covered by a gold film. The enhancement in that Raman signal that is about 10 times that seen previously in Figure 4b could be interpreted by synergistic plasmonic effects. Contrary to the silicon sample, the dielectric feature of the silk substrate and its combination with the gold nanocracks was more relevant for an efficient excitation of the localized surface plasmon modes. Additionally, a narrow gap distance was beneficial in generating hot-spot modes in the gold nanocracks, leading to an enormous signal amplification.

**Figure 5:**
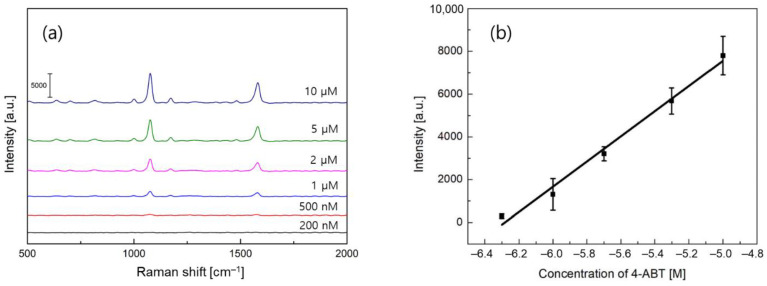
SERS sensor performance measurement. (**a**) SERS experiments of 4-ABT analytes at a varied concentrations for a silk substrate with gold nanocracks. (**b**) Linear regression analysis of the Raman intensities at 1076 cm^−1^ to quantify the sensitivity and linearity.

Next, we evaluated the linearity performance between SERS peak intensity and concentration of 4-ABT. For the silk samples with gold nanocracks, the detection limit was experimentally obtained to be 500 nM in Figure 5a, and the sensitivity and *R*^2^ values of 5890 and 0.96 were determined from the linear regression data in Figure 5b. The linearity was also quite good in a wide dynamic range. It is very interesting to note that compared to the results of silk samples with a 40 nm thick gold film in Figure 3, both the detection limit and sensitivity characteristics were improved by about four times. In order to understand the contrast in the performance of silk samples with a gold film or gold nanocracks, in-depth investigations were necessary.

**Figure 6:**
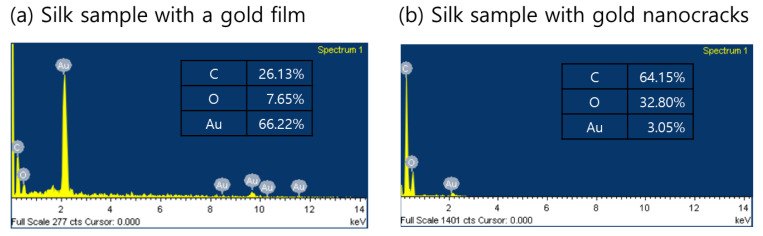
Energy-dispersive X-ray spectroscopy measurements. Percentage weight of contents for (**a**) porous silk-based SERS sensor with a 40 nm thick gold film and (**b**) porous silk-based SERS sensor with a 3 nm thick gold nanocracks.

Figure 6 presents the composition analyses of two silk-based SERS substrates determined using energy-dispersive X-ray spectroscopy (EDS) measurements. Hydrogen (H) and nitrogen (N) contents of silk fibroin were not detected due to the small atomic mass of hydrogen and the extremely low responsivity of nitrogen [29]. Obviously, the percentage content of gold was drastically increased as a result of gold deposition between the two silk samples. For the 40 nm thick gold film, there was relative increment in the percentage weight of the gold that was more than 20-fold. Such a difference in the amount of gold would be responsible for the formation of gold nanocracks and the excitation of plasmon fields.

In addition, we studied the underlying physics of localized field amplification and plasmonic field-analyte interaction using the finite-element method (FEM). FEM models were prepared for silk substrates with flat and non-flat surface profiles. Refractive indices (*n*, *k*) of gold and silk at λ = 785 nm were chosen to be (0.2313, 4.3933) and (1.5405, 0), respectively [30,31]. As a quantitative metric of the field–analyte interactions, we defined an overlap integral (OI), which is the integration of local field intensity within a 0.2 nm thick binding layer of 4-ABT [32,33]. Illumination was assumed as a normal incidence of a unit amplitude. For non-flat silk samples, a rough surface profile was designed using the real AFM data in Figure 1c, whose surface roughness was 38.55 nm. The overall surface dimension of 1 μm was large enough for the given geometric values of a 40 nm thick gold film and 3 nm thick gold nanocracks, so a number of statistical variations were included in a constructed surface profile, indicating that the use of a single rough surface could provide valid insights on the roughness effect.

**Figure 7:**
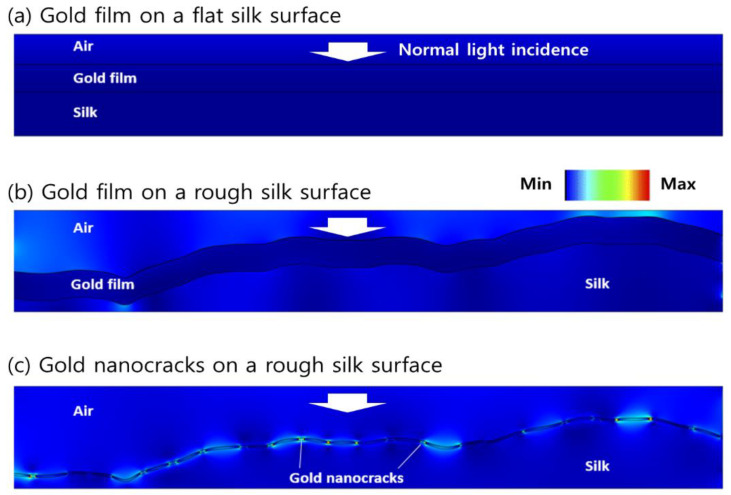
Numerical analyses on local field enhancement using FEM method. Field distributions of SERS substrate for (**a**) a 40 nm thick gold film on a flat silk substrate, (**b**) a 40 nm thick gold film on a rough silk substrate, and (**c**) a 3 nm thick gold nanocracks on a rough silk substrate. FEM models of rough silk surface were prepared using real AFM data with a surface roughness of 38.55 nm. Monochromatic light of λ = 785 nm was assumed as a normal incidence of a unit amplitude.

Among three FEM results, a 40 nm thick gold film on a flat silk substrate, known as a typical surface plasmon resonance configuration, showed no significant field amplification in Figure 7a. Under a normal incidence condition, the electromagnetic fields near the gold surface were not enhanced nor localized. The peak field amplitude was 1.22, and the OI value that was obtained was 1.51 × 10^4^. On the other hand, a thin gold film with a non-flat silk surface that displayed a fairly enhanced field at several protrusions on the gold surface, as presented in Figure 7b. While the excited fields were not highly localized, the field amplification was quantitatively obtained to be 6.79 for a peak field amplitude and 2.94 × 10^5^ for an OI value, respectively. It is reasonable to mention that such an increase of the field amplitude and OI value led to procurable SERS signals in Figure 2c. For gold nanocracks on a rough silk substrate, a strong enhancement of the local plasmon field was found between the gold nanocracks. When the summation of the field intensity over the surface was calculated, a maximum field amplitude of 51.12 and an OI value of 1.21 × 10^6^ were determined. It is also noteworthy that the OI enhancement of 4.11 times by the gold nanocracks was consistent with the improvement of the detection limit and sensitivity by about 4 times, as seen in Figure 3b and Figure 5b. Moreover, we speculate that the non-uniform plasmon field distribution of the gold nanocracks on the porous silk substrate in Figure 7c will not deteriorate the reliability of SERS detection. While the results were not shown here, the total sum of the field amplitudes over the sensing surface dimension of 10 μm presented a good standard deviation of 10% compared to its average value. Based on the good correlation between the OI and SERS characteristics, FEM analyses demonstrated that the combination of the rough and porous silk substrate and gold nanocracks could provide a higher detection performance via an efficient excitation of the localized plasmon fields, expanding applications to a variety of fields, such as noninvasive diagnostics and healthcare monitoring.

Finally, in terms of the biocompatibility of the proposed SERS scheme, the biological responses of silk have been extensively explored, revealing its excellent bio-response in vivo with low immunogenicity [34]. It is evident from previous publications that the combination of silk and gold nanostructures did not exhibit any cytotoxicity in vitro and in vivo [35,36,37,38,39]. However, to verify this issue more specifically, we intend to evaluate the biocompatibility in our device in the subsequent works.

## 4. Conclusions

In this study, we reported the fabrication and characterization of a novel SERS sensor consisting of a porous silk substrate and gold nanostructures. The target analytes of 4-ABT adsorbed on the sensor surface experienced the SERS effect and led to high sensitivity through an interaction between the local plasmon fields and analytes. The experimental data showed that the porous silk samples with a 40 nm thick gold film verified the effect of the porous sensor surface, and, more importantly, the combination with gold nanocracks demonstrated a hot-spot effect at the nanoscale gap regions, finally resulting in an improvement of about 4 times in the detection limit and the sensitivity as well as in good linearity and dynamic range characteristics. Moreover, a numerical investigation based on FEM showed that the proposed SERS sensor was effective for amplifying the local plasmon fields and encouraging a field–analyte interaction. Our silk-based SERS sensor platform with biocompatibility could be available for the highly sensitive monitoring of various analytes for in vitro and in vivo applications.
